# CaMKII Exacerbates Doxorubicin‐Induced Cardiotoxicity by Promoting Ubiquitination Through USP10 Inhibition

**DOI:** 10.1002/cam4.70286

**Published:** 2024-11-08

**Authors:** Yitong Yang, Zhenyi Wang, Nisha Wang, Jian Yang, Lifang Yang

**Affiliations:** ^1^ Department of Anesthesiology Children's Hospital of Xi'an Jiaotong University Xi'an Shaanxi China; ^2^ Department of Children's Respiratory Asthma Second Affiliated Hospital of Shaanxi University of Chinese Medicine Xianyang Shaanxi China; ^3^ Department of Cardiovascular Surgery Xijing Hospital, Air Force Medical University Xi'an China

**Keywords:** CaMKII, cardiotoxicity, doxorubicin, H9C2, ubiquitination, USP10 pathway

## Abstract

**Background:**

Doxorubicin (DOX) is an effective anticancer drug, but it has a problem of cardiotoxicity that cannot be ignored. Ca^2+^/calmodulin‐dependent protein kinase II (CaMKII) is tightly associated with the pathological progression of DOX‐induced cardiotoxicity. Ubiquitin‐specific protease 10 (USP10) plays an important role in many biological processes and cancers. However, its association with DOX‐induced cardiotoxicity and CaMKII remains unclear.

**Methods:**

H9C2 cells, HL‐1 cells and C57BL/6 mice were used to establish the DOX‐induced cardiotoxicity model, and the CaMKII‐specific inhibitor KN‐93 and USP10 specific inhibitor Spautin‐1 were used to observe the CaMKII and USP10 effect. In cell experiments, CCK‐8 method was used to assess cell viability, LDH kit was used to assess lactate dehydrogenase expression, DCFH‐DA staining was used to observe changes in active oxygen content, TUNEL staining was used to observe cell apoptosis, and Western blotting method was used to detect relevant protein markers. The expression of p‐CaMKII and USP10 was assessed by immunofluorescence staining. In animal experiments, mouse echocardiograph was used were used to evaluate cardiac function, and HE staining and Masson staining were used to evaluate myocardial injury. Cardiomyocyte apoptosis was detected by TUNEL staining. Western blotting method was used to detect relevant protein markers.

**Results:**

Our results demonstrated that activation of CaMKII and inhibition of USP10 pathway related to DOX‐induced cardiotoxicity. Inhibition of CaMKII with KN‐93 ameliorated DOX‐induced cardiac dysfunction and cytotoxicity. In addition, CaMKII inhibition prevented DOX‐induced apoptosis and ubiquitination. Furthermore, CaMKII inhibition increased USP10 expression in DOX‐treated mouse hearts, H9C2 cells and HL‐1 cells. At last, the USP10 inhibitor, Spautin‐1, blocked the regulatory effect of CaMKII inhibition on apoptosis and ubiquitination in DOX‐induced cardiotoxicity.

**Conclusion:**

Our findings revealed that DOX‐induced myocardial apoptosis and activated CaMKII through cellular and animal levels, while providing a novel probe into the mechanism of CaMKII action: promoting ubiquitination by inhibiting USP10 aggravated apoptosis.

## Graphical abstract

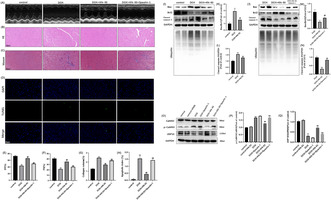

## 1 Introduction

Doxorubicin (DOX) is a commonly prescribed anticancer medication that inhibits the growth of several tumor types, including malignant lymphoma, breast cancer, and bronchopulmonary cancer [1, 2]. It is an important clinical antitumor drug that exhibits high toxicity toward normal cells. The considerable cardiotoxicity associated with DOX cannot be ignored [3, 4]. Typical manifestations include left ventricular systolic‐diastolic dysfunction, eccentric ventricular hypertrophy, and heart failure [5, 6]. Currently, studies on the primary mechanisms of DOX‐induced cardiotoxicity focus on disrupting mitochondrial function [7], increasing oxidative stress [8, 9], autophagic dysfunction [10], and promoting apoptosis [11]. However, the underlying pathological mechanism of DOX‐induced cardiotoxicity remains elusive, making it essential to continue investigating its mechanism.

Ubiquitin is a small molecule regulatory protein that is ubiquitous in eukaryotes. In the human genome, four genes encode ubiquitin proteins: UBB, UBC, UBA52 and RPS27A. Ubiquitination is the biochemical process through which ubiquitin proteins attach to precise sites on substrate protein molecules, while deubiquitination refers to the removal of ubiquitin molecules. These processes play crucial roles in cell physiology, particularly in the targeted degradation of proteins and the modulation of cell signaling pathways [12]. Ubiquitination modifies specifically targeted proteins and plays a crucial role in the regulation of almost all the cellular activities [13, 14]. It impacts protein localization, metabolism, function, regulation, and degradation [15, 16]. Deubiquitinating enzymes are responsible for the specific hydrolysis of ubiquitin molecules from the protein or precursor protein linked with ubiquitin by hydrolyzing the ester bond, peptide bond or isopeptide bond at the carboxyl terminal of ubiquitin, which plays the role of deubiquitination and counterregulates protein degradation, thereby affecting protein function [17]. Ubiquitin‐specific protease 10 (USP10) functions as a cysteine hydrolase that hydrolyzes the ester bond between the glycine at the carboxyl terminus of ubiquitin and the lysine of the substrate. This enzymatic activity enables the removal of ubiquitin from target proteins, allowing USP10 to perform several functions at the cellular level. USP10 plays an important role in many biological processes and cancers. And regulating the USP10 signaling pathway can impact the regulation of apoptosis and injury in cardiac cells [18]. Studies have found that USP10 acts as a deubiquitinase of Sirt6 to induce cardiomyocyte hypertrophy and trigger cardiac hypertrophy, and cardiac fibroblasts could aggravate myocardial ischemia–reperfusion fibrosis by enhancing USP10‐dependent Smad4 deubiquitination.

In addition, FoxO4 can aggravate cardiomyocyte apoptosis and oxidative stress by regulating USP10 transcription [18, 19, 20]. These results suggest that USP10 plays an important role in the progression of heart injury. And abnormal ubiquitination and apoptosis are closely associated with the development of tumors and cardiovascular disorders among other diseases [21, 22]. However, whether USP10 can regulate DOX‐induced cardiotoxicity remains unclear.

Calcium/calmodulin‐dependent protein kinase II (CaMKII) is a kinase that exhibits high expression levels in cardiac tissue. It accelerates myocardial diastolic process in physiological condition and plays an important role in maintaining intracellular calcium homeostasis [23, 24]. Ca^2+^ plays a key regulatory role in the function and metabolic activities of cardiomyocytes. It is not only an important factor of excitation‐contraction coupling, but also participates in the regulation of cell apoptosis as the second messenger. On the one hand, CaMKII, as an important protein kinase in the body, increases the sensitivity of Ryanodine receptor 2 (RyR2) to Ca^2+^ and the release of Ca^2+^ from sarcoplasmic reticulum calcium stores by phosphorylating RyR2. On the other hand, the phosphorylation of phospholamban (PLB) could relieve the inhibitory effect of Sarcoplasmic/endoplasmic reticulum Ca^2+^ ATPase 2a (SERCA2a) and enhance the Ca^2+^ uptake of sarcoplasmic reticulum. However, excessive activation of CaMKII lead to impaired cardiac function [25]. CaMKII is involved in several processes including cardiomyocyte apoptosis, cardiac hypertrophy, heart failure, arrhythmias, and dilated cardiomyopathy [26]. It is worth noting that studies have shown that CaMKII is closely related to DOX‐induced cardiotoxicity and regulation of calcium homeostasis through CaMKII is a potential therapeutic mechanism to alleviate DOX‐induced cardiotoxicity [27, 28]. In the process of cardiomyocyte apoptosis, calcium first promotes the phosphorylation of CaMKII, and activated CaMKII further promotes the increase of ROS level and apoptosis. At the same time, apoptosis is closely related to ubiquitination, and ubiquitination modification of apoptotic proteins plays a huge role in cell death signaling pathways that cannot be ignored. It has been published that activation of ubiquitination leads to dysregulation of CaMKII, which results in calcium overload and exacerbates DOX‐induced cardiotoxicity [29]. However, it remains unclear whether CaMKII mediates DOX‐induced cardiotoxicity through the regulation of deubiquitinating enzyme USP10 to promote ubiquitination and further aggravate apoptosis.

This study aimed to investigate the in vivo and in vitro effects of DOX‐induced cardiotoxicity. We further investigated the effect of CaMKII on ubiquitination and apoptosis in DOX‐induced cardiotoxicity using the CaMKII‐specific inhibitor KN‐93. Subsequently, we used the USP10‐specific inhibitor Spautin‐1 to explore the mechanism by which CaMKII aggravated the DOX‐induced cardiotoxicity by inhibiting USP10 signaling to promote ubiquitination and aggravate apoptosis.

## 2 Materials and Methods

### 2.1 Reagents

Antibodies against ubiquitin, USP10, USP13, Bcl2, Bax, and GAPDH were purchased from Proteintech (Chicago, IL, USA). The anti‐CaMKII antibody was purchased from Gene Tex (San Antonio, USA), while the p‐CaMKII antibody was obtained from Abcam (Cambridge, UK), and the Cleaved Caspase‐3 antibody was obtained from CST (Boston, USA). Additionally, the Pierce BCA protein assay kit was purchased from Thermo Scientific (Waltham, MA, USA). DOX and the Micro Mitochondrial Respiratory Chain ComplexIActivity Assay Kit were obtained from SolarBio (Beijing, China). KN‐93 and Spautin‐1 were purchased from MedChemExpress (New Jersey, USA). The Reactive Oxygen Species Assay Kit was sourced from Beyotime Biotechnology (Shanghai, China). The Enhanced Cell Counting Kit 8, Lactate Dehydrogenase Activity Assay Kit, and one‐step TUNEL In Situ Apoptosis Kit were purchased from Elabscience (Wuhan, China). Additionally, the goat anti‐rabbit and goat anti‐mouse secondary antibodies were obtained from Zhongshan Company (Beijing, China).

### 2.2 Cells and Experimental Protocols

The H9C2 and HL‐1 myocardial cell lines was procured from the Cell Bank of the Type Culture Collection of the Chinese Academy of Sciences. Subsequently, the cells were maintained in Dulbecco's Modified Eagle Medium supplemented with 1% penicillin–streptomycin and 10% fetal bovine serum. The experimental protocol involved incubating cells at 37°C in a 5% CO_2_ and 95% O_2_ atmosphere. Cells were then subjected to specific treatments using KN‐93 (1 μM) or Spautin‐1 (10 μM) for a duration of 12 h, followed by treatment with DOX (1 μM) for 24 h. The selection of drug concentrations for this study was guided by prior research findings [30, 31].

### 2.3 Animals and Experimental Protocols

Adult male C57BL/6J mice, weighing between 25 and 30 g, were obtained from the Laboratory Animal Center at the Fourth Military Medical University in Xi'an, China. The research protocols conducted in this study were ethically approved by the Ethics Committee of the Fourth Military Medical University (approval no: 20220431). C57BL/6J mice were randomly assigned to four different groups: control, DOX, DOX + KN‐93, and DOX + KN‐93 + Spautin‐1. The mice in the DOX group received intraperitoneal injections of DOX (15 mg/kg) once, as well as intraperitoneal injections of KN‐93 (5 mg/kg) and Spautin‐1 (2 mg/kg) the day before the initial DOX treatment. All groups had a 7‐day processing period. The dosage of medication administered in this research was determined by referencing prior studies [32, 33]. The mice were closely observed, and their well‐being was documented on a daily basis.

### 2.4 Echocardiography and Hemodynamic Measurement

C57BL/6J mice were sedated with 2% isoflurane 1 week post DOX administration for cardiac assessment. The examination involved short‐axis measurements utilizing two‐dimensional and M‐mode imaging with a Vevo 2100 ultrasound system from VisualSonics in Canada. Subsequently, the gathered images were scrutinized to evaluate different cardiac function metrics such as ejection fraction (EF) and fractional shortening (FS) using Vevo software version 5.6 from Bothell, USA.

### 2.5 Histopathological Examination

The heart tissues were rinsed with pre‐cooled PBS, followed by fixation in 4% paraformaldehyde at room temperature for a duration of 24 h. Subsequently, the tissues were encased in paraffin for histological examination. Evaluation of heart morphology and assessment of myocardial fibrosis were carried out through Hematoxylin and Eosin (HE) staining, as well as Masson's trichrome staining.

### 2.6 Cell Viability

H9C2 cells were plated in 96‐well plates following the specified treatments for each group. Cell viability was evaluated using a CCK‐8 assay, where 10 μL of CCK‐8 solution was applied to the cells and incubated for 1.5 h. The assessment of cell viability was based on the optical density comparison between each group and the control cells.

### 2.7 Cell LDH Viability

LDH levels were assessed utilizing the LDH kit following the strict guidelines provided by the manufacturer, with calculations executed accordingly.

### 2.8 DCFH‐DA Staining

H9C2 cells were cultured in confocal dishes and subjected to ROS analysis through DCFH‐DA staining post‐treatment. The cells were incubated with 10 μM DCFH‐DA at 37°C for 30 min. Fluorescent images were then obtained utilizing an Olympus Fluoview FV 1000 microscope (Olympus, Japan), followed by quantification of fluorescence intensity via ImageJ software (version 6.0, NIH, Germany).

### 2.9 Terminal Deoxynucleotidyl Transferase dUTP Nick End Labeling (TUNEL) Assay

The one‐step TUNEL In Situ Apoptosis Kit was utilized to label cardiomyocytes in frozen sections and H9C2 cells subsequent to fixation with 4% paraformaldehyde and permeabilization with 0.2% Triton X‐100. Subsequent to the preparation, TUNEL and DAPI staining procedures were conducted. The fluorescent images were visualized utilizing an Olympus Fluoview FV1000 microscope (Olympus, Japan).

### 2.10 Mitochondrial Complex I Activity Assay

The activities of Mitochondrial complex I were measured and analyzed using the Micro Mitochondrial Respiratory Chain ComplexIActivity Assay Kits, following the manufacturer's instructions. The absorbance was measured at 340 nm on an enzyme labeler.

### 2.11 Immunofluorescence Staining

Immunofluorescence staining was performed to evaluate the presence of p‐CaMKII and USP10 in H9C2 cells. Following the specified treatments, H9C2 cells were cultured in confocal dishes. The cells were fixed with 4% paraformaldehyde for 1 h and permeabilized with 0.2% Triton X‐100 for 15 min. Subsequently, primary antibodies targeting p‐CaMKII or USP10 were applied to the cells overnight at 4°C. After incubation, the cells were treated with fluorescent secondary antibodies for 1 h at 37°C and stained with DAPI for 30 min at the same temperature. The fluorescent images were captured using an Olympus Fluoview FV1000 microscope (Olympus, Japan). Finally, the fluorescence intensity was analyzed using ImageJ software (version 6.0, NIH, Germany).

### 2.12 Western Blotting

The supernatant obtained post lysing of H9C2 cells and myocardial tissues using RIPA buffer was collected. The protein concentration was assessed using the BCA method, followed by separation of proteins via SDS‐PAGE and transfer onto PVDF membranes. Blocking of membranes was carried out with 5% nonfat skim milk for 2 h at room temperature. Subsequently, bands corresponding to molecular sizes were excised and incubated overnight at 4°C with specific primary antibodies. The following day, membranes were treated with respective secondary antibodies for 2 h. Chemiluminescence reagents were utilized for detecting antigen–antibody complexes, and visualization of bands was performed using a ChemiDoc XRS system (Bio‐Rad, Hercules, USA). GAPDH served as the internal control.

### 2.13 Statistical Analysis

The experimental data underwent statistical analysis through GraphPad Prism 9.5 software (version 8.0; GraphPad Software, San Diego, CA, USA). Results are expressed as mean ± SEM. Statistical evaluation included a one‐way analysis of variance, succeeded by Tukey post hoc comparisons. Significance was determined at *p* < 0.05.

## 3 Results

### 3.1 CaMKII Inhibition With KN‐93 Alleviated DOX‐Induced H9C2 Injury and Apoptosis

**FIGURE 1:**
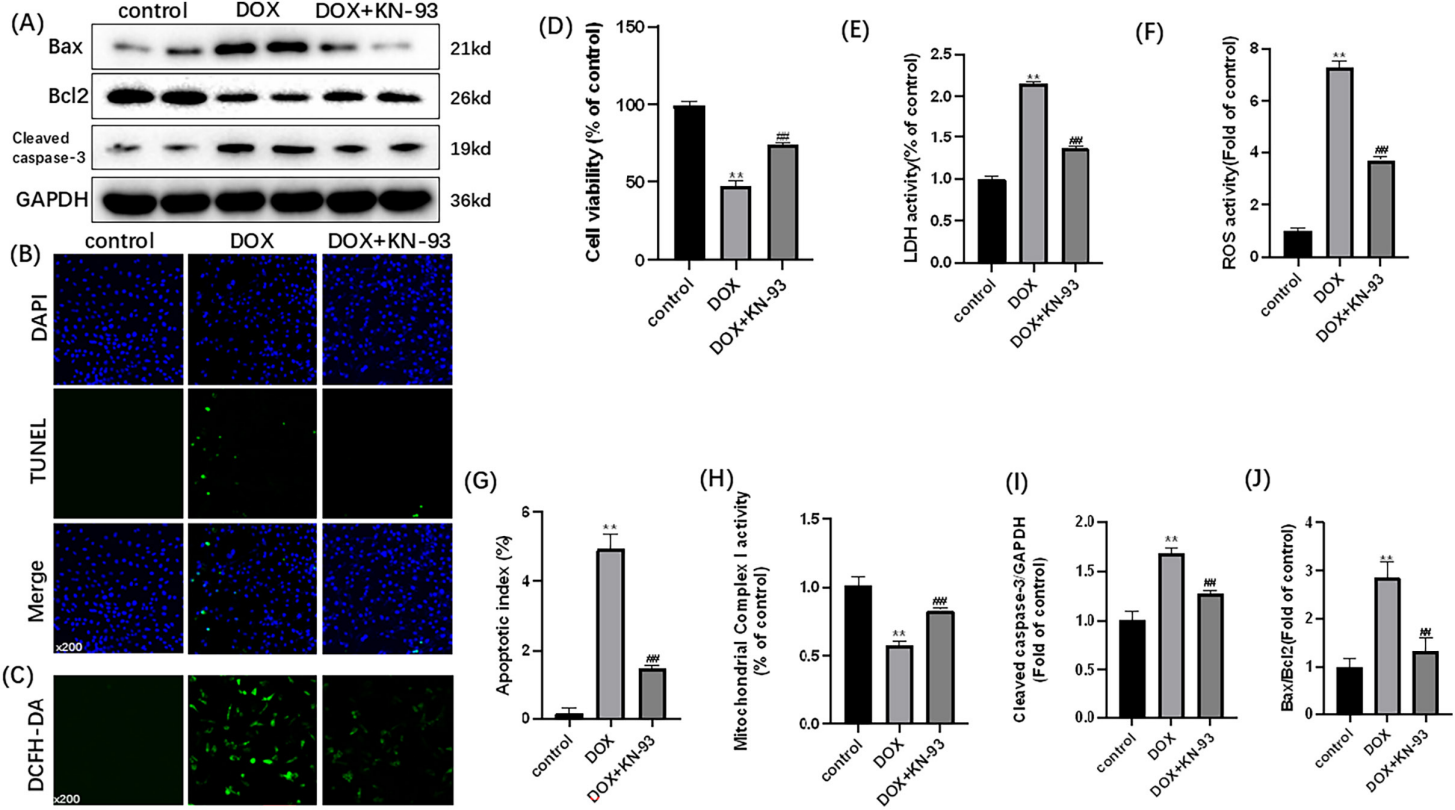
CaMKII inhibition with KN‐93 alleviates DOX‐induced H9C2 injury and apoptosis. Representative images of (A) immunoblots of Bax, Bcl2, cleaved caspase‐3 and GAPDH; (B) TUNEL staining (×200); (C) DCFH‐DA staining (×200). (D) Cell viability; (E) LDH activity; (F) ROS activity; (G) apoptotic index; (H) mitochondrial complex I activity. (I) Statistical analysis of Cleaved Caspase‐3/GAPDH. (J) Statistical analysis of Bax/Bcl2. The data are represented as mean ± SEM, *n* = 3–6. Statistical analysis was performed using a one‐way analysis of variance, followed by Tukey post hoc tests. **p < 0.05 versus control, ^##^ *p* <  0.05 versus DOX.

To observe the effects of DOX on cardiomyocytes, we used DOX‐treated H9C2 cells. As depicted in Figure 1, DOX treatment resulted in reduced cell viability and mitochondrial complex I activity, an increase in LDH activity, apoptotic index, ROS activity (Figure 1B–H), Cleaved Caspase‐3/GAPDH and Bax/Bcl2 ratio in H9C2 cardiomyocytes (Figure 1A,I,J). When H9C2 cells were treated with KN‐93, cell viability increased, while LDH activity, apoptotic index, ROS activity (Figure 1B–H), and the Bax/Bcl2 ratio decreased (Figure 1A,I,J). These findings support the notion that DOX can induce damage and apoptosis in H9C2 cells, which can be alleviated by inhibiting CaMKII with KN‐93.

### 3.2 CaMKII Inhibition With KN‐93 Alleviated DOX‐Induced H9C2 Ubiquitination

**FIGURE 2:**
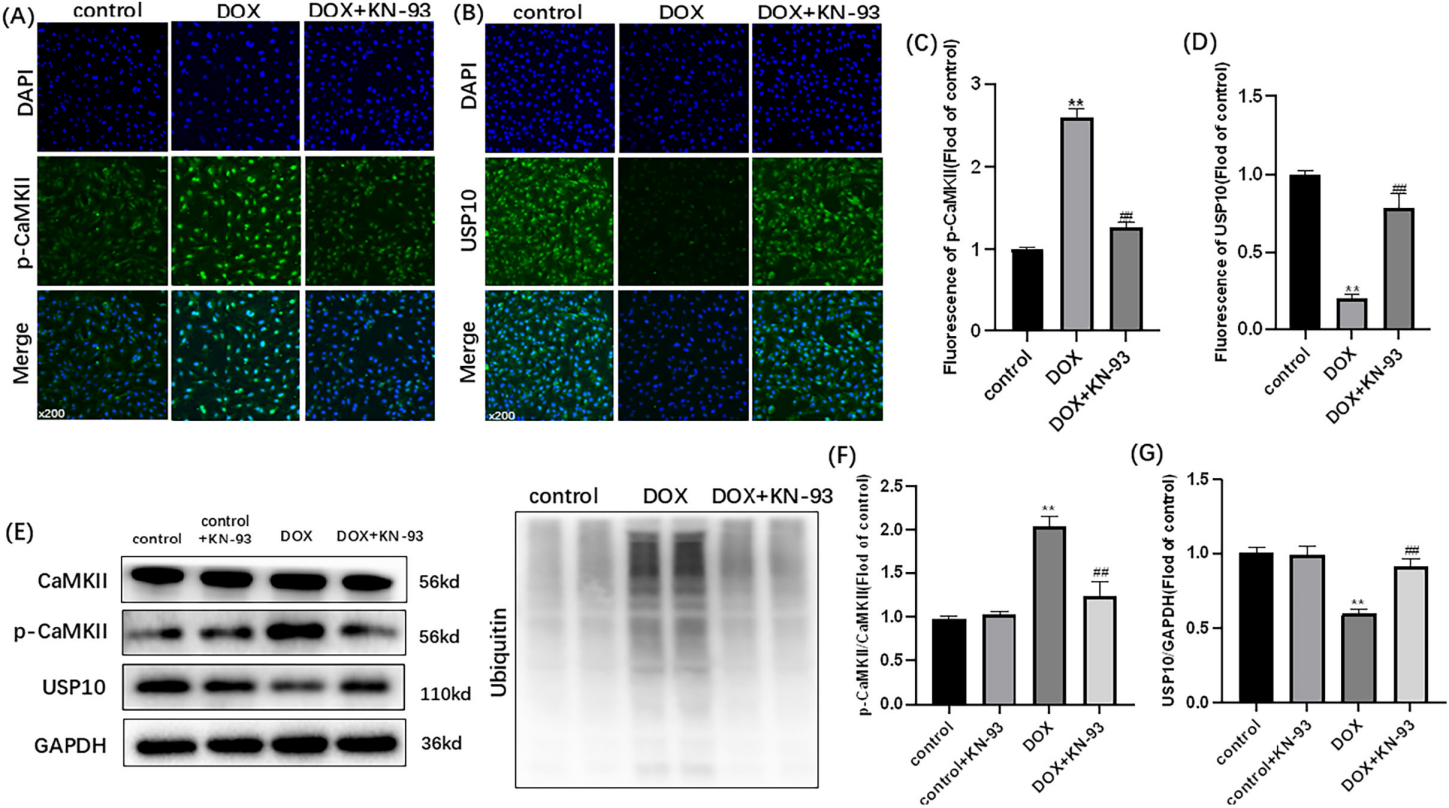
CaMKII inhibition with KN‐93 alleviates DOX‐induced H9C2 ubiquitination. Representative images of (A) p‐CaMKII staining (×200); (B) USP10 staining (×200); Fluorescence intensity of (C) p‐CaMKII; (D) USP10. (E) Representative immunoblots of CaMKII, p‐CaMKII, USP10, GAPDH and ubiquitin. Statistical analysis of (F) p‐CaMKII/CaMKII; (G) USP10. The data are represented as mean ± SEM, *n* = 3–6. Statistical analysis was performed using a one‐way analysis of variance, followed by Tukey post hoc tests. **p < 0.05 versus control, ^##^ *p* <  0.05 versus DOX.

As shown in Figure 2, DOX‐induced ubiquitination in H9C2 cells, as evidenced by increased expression of ubiquitin and p‐CaMKII, and decreased expression of USP10 (Figure 2E–G). The immunofluorescence staining of p‐CaMKII and USP10 aligned with our expected changes (Figure 2A–D). Following treatment with KN‐93, the expression of USP10 and the immunofluorescence intensity of USP10 increased significantly, while those of ubiquitin and p‐CaMKII, and the immunofluorescence intensity of p‐CaMKII were decreased (Figure 2A–G). CaMKII exacerbated DOX‐induced H9C2 cell injury through ubiquitination, and inhibiting CaMKII alleviate H9C2 cell ubiquitination. In conclusion, the expression of CaMKII was increased in DOX‐induced H9C2 cells, leading to ubiquitination and apoptosis. However, inhibition of CaMKII with KN‐93 not only reduced the degree of injury but also markedly reduced ubiquitination and apoptosis. These findings suggest that CaMKII is closely related to DOX‐induced cardiotoxicity, and USP10 may play an important role in this process, as supported by western blotting and immunofluorescence staining results.

### 3.3 Spautin‐1 Blocked the Protective Effects of CaMKII Inhibition on DOX‐Induced H9C2 Injury and Apoptosis

**FIGURE 3:**
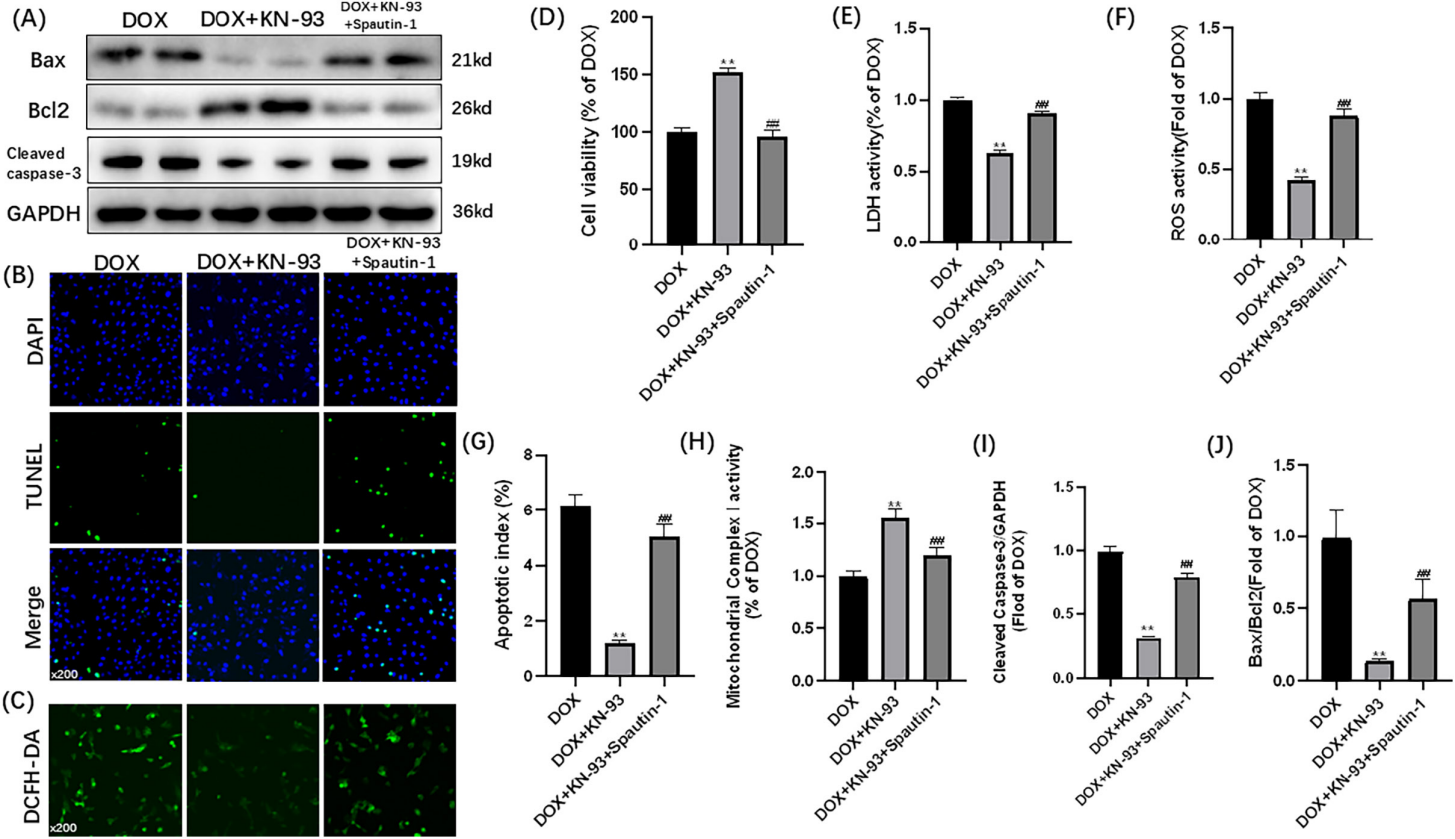
Spautin‐1 blocks the protective effects of CaMKII inhibition on DOX‐induced H9C2 injury and apoptosis. Representative images of (A) immunoblots of Bax, Bcl2, Cleaved Caspase‐3 and GAPDH; (B) TUNEL staining (×200); (C) DCFH‐DA staining (×200). (D) Cell viability; (E) LDH activity; (F) ROS activity; (G) Apoptotic index; (H) Mitochondrial Complex I activity. (I) Statistical analysis of Cleaved Caspase‐3/GAPDH. (J) Statistical analysis of Bax/Bcl2. The data are represented as mean ± SEM, *n* = 3–6. Statistical analysis was performed using a one‐way analysis of variance, followed by Tukey post hoc tests. **p < 0.05 versus DOX, ^##^ *p* <  0.05 versus DOX + KN‐93.

To further verify whether USP10 is the pathway of KN‐93 action, we employed Spautin‐1's effects in H9C2 cells. As illustrated in Figure 3, compared to the DOX + KN‐93 group, the DOX + KN‐93 + Spautin‐1 group exhibited reduced cell viability and mitochondrial Complex I activity, along with an increase in LDH activity, apoptosis index, ROS activity (Figure 3B–H), Cleaved Caspase‐3/GAPDH, and the Bax/Bcl2 ratio (Figure 3A,I,J). The protective effect of CaMKII inhibition on DOX‐induced injury and apoptosis in H9C2 cells was abolished upon intervention with Spautin‐1. This strongly suggests that the USP10 signaling pathway plays a crucial role in mediating the effectiveness of KN‐93.

### 3.4 Spautin‐1 Blocked the Protective Effects of CaMKII Inhibition on DOX‐Induced H9C2 Ubiquitination

**FIGURE 4:**
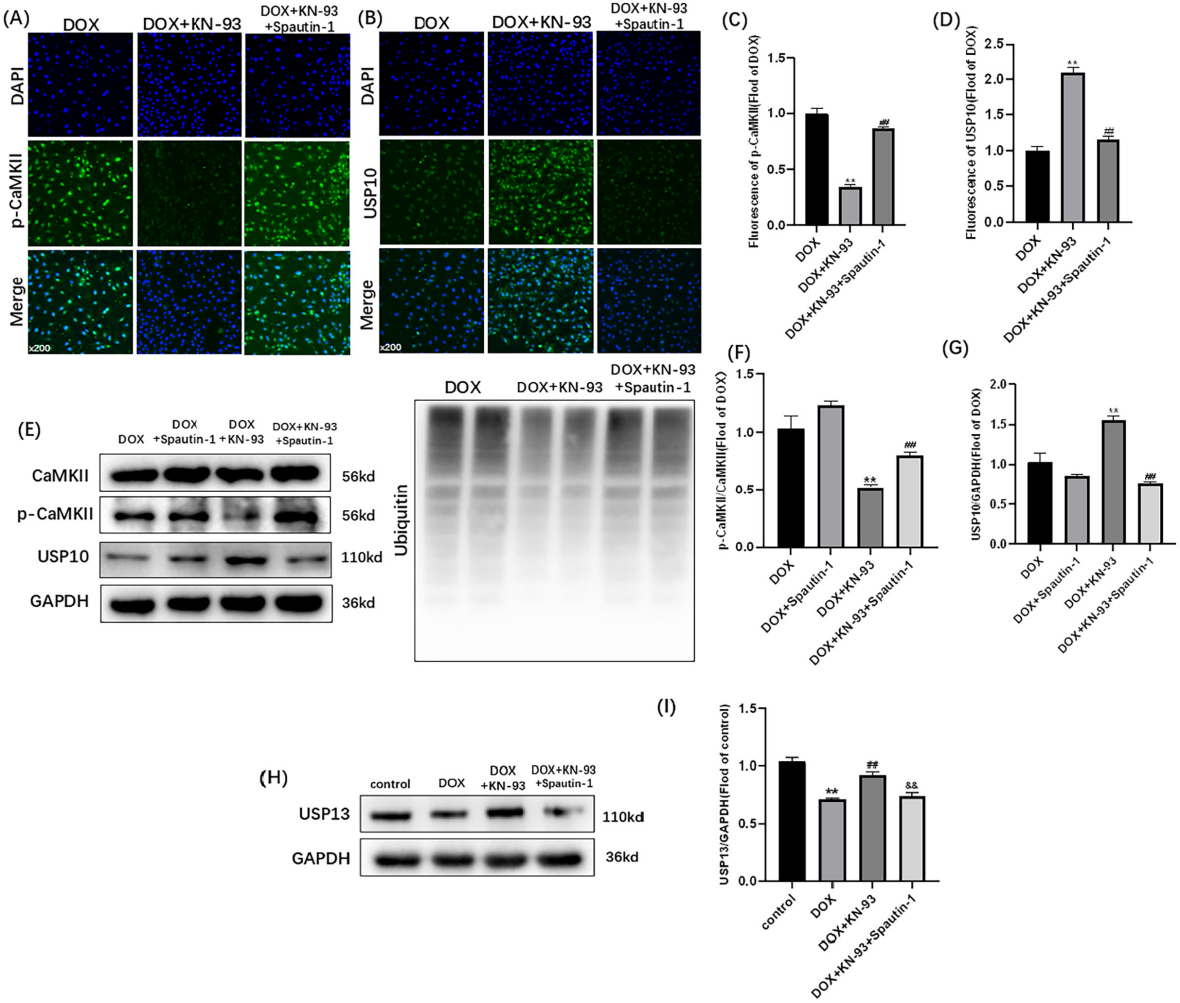
Spautin‐1 blocks the protective effects of CaMKII inhibition on DOX‐induced H9C2 ubiquitination. Representative images of (A) p‐CaMKII staining (×200); (B) USP10 staining (×200); Fluorescence intensity of (C) p‐CaMKII; (D) USP10. (E) Representative immunoblots of CaMKII, p‐CaMKII, USP10, GAPDH and ubiquitin. Statistical analysis of (F) p‐CaMKII/CaMKII; (G) USP10; (H) Representative immunoblots of USP13. (I) Statistical analysis of USP13. The data are represented as mean ± SEM, *n* = 3–6. Statistical analysis was performed using a one‐way analysis of variance, followed by Tukey post hoc tests. (A–G, **p < 0.05 vs. DOX, ^##^ *p* <  0.05 vs. DOX + KN‐93); (H, I, **p < 0.05 vs. control, ^##^ *p* <  0.05 vs. DOX, ^&&^ *p* <  0.05 vs. DOX + KN‐93).

Upon analyzing the data presented in Figure 4, a comparison was made between the DOX + KN‐93 group and the DOX + KN‐93 + Spautin‐1 group. In the DOX + KN‐93 + Spautin‐1 group, there was a decrease in the expression of USP10, along with a reduction in USP10 immunofluorescence. Conversely, there was an increase in the expression of ubiquitin and p‐CaMKII, along with an increase in p‐CaMKII immunofluorescence (Figure 4A–G). The protective effect of CaMKII inhibition on DOX‐induced H9C2 ubiquitination was nullified with the use of Spautin‐1. It strongly suggests that CaMKII exacerbates DOX‐induced cardiotoxicity by modulating ubiquitination through the USP10 pathway. In addition, Spautin‐1 can also inhibit USP13, so we detected the expression of USP13. We found that myocardial cell damage induced by DOX can decrease the expression of USP13, while KN‐93 can increase the expression of USP13. And Spautin‐1 can block KN‐93 from increasing USP13 expression in DOX‐induced cardiomyocyte injury (Figure 4H,I). This suggests that USP13 may also be a regulatory molecule of CaMKII, and we will explore this mechanism in further studies.

### 3.5 The Changes of CaMKII‐USP10 in DOX‐Induced HL‐1 Cardiomyocyte Injury Were the Same as the H9C2 Cell Line

**FIGURE 5:**
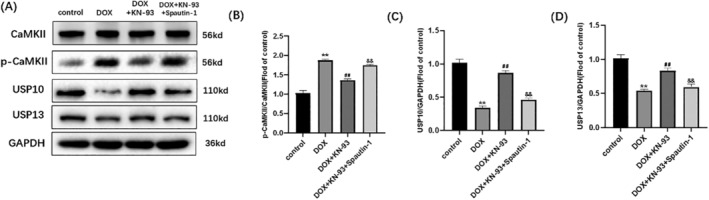
The changes of CaMKII‐USP10 in DOX‐induced HL‐1 cardiomyocyte injury were the same as the H9C2 cell line. (A) Representative immunoblots of CaMKII, p‐CaMKII, USP10, USP13 and GAPDH; (B–D) Statistical analysis of p‐CaMKII/CaMKII, USP10/GAPDH, USP13/GAPDH. **p < 0.05 vs. control, ^##^ *p* <  0.05 vs. DOX, ^&&^ *p* <  0.05 versus DOX + KN‐93. The data are represented as mean ± SEM, *n* = 3–6. Statistical analysis was performed using a one‐way analysis of variance, followed by Tukey post hoc tests.

The role of CaMKII‐USP10 in DOX‐induced cardiotoxicity was further validated using the HL‐1 cardiomyocyte. As shown in Figure 5, In HL‐1 cells, compared with the control group, the expression of p‐CaMKII was significantly increased in DOX group, and the expression of USP10 and USP13 was significantly decreased, while the expression of p‐CaMKII and USP10 and USP13 were significantly increased in DOX + KN‐93 group compared with DOX group. After Spautin‐1 intervention, compared with DOX + KN‐93 + Spautin‐1 group, p‐CaMKII expression was significantly increased, while USP10 and USP13 expression was significantly decreased in DOX + KN‐93 + Spautin‐1 group. This is consistent with our findings in H9C2 cells, which together demonstrate the role of CaMKII in regulating USP10 in DOX‐induced cardiotoxicity.

### 3.6 CaMKII Inhibition With KN‐93 Alleviated DOX‐Induced Cardiac Function and Structure Changes, and Spautin‐1 Blocked the Protective Effects

**FIGURE 6:**
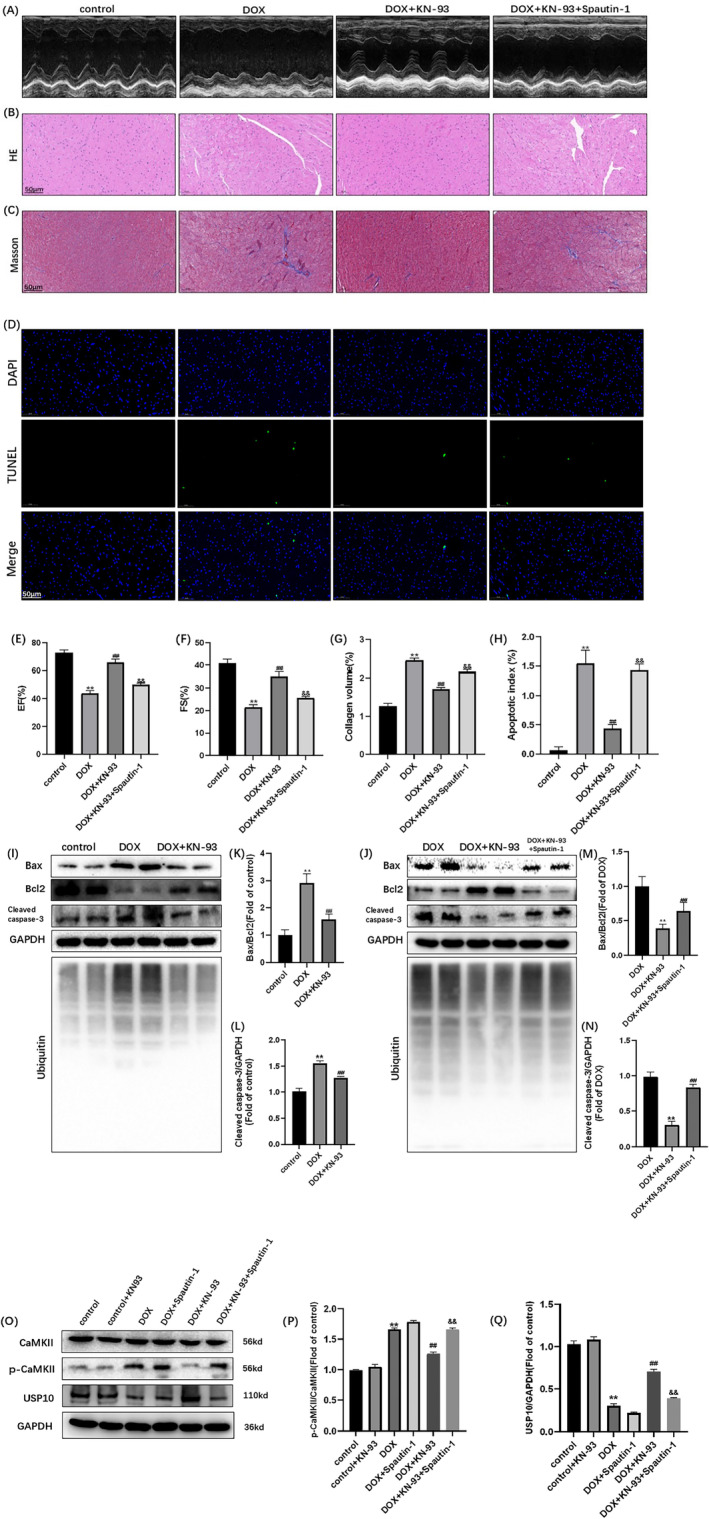
CaMKII exacerbates dox‐induced mice myocardial injury by promoting ubiquitination and apoptosis through USP10 signaling inhibition, and Spautin‐1 blocks the protective effect of KN‐93. Representative images of (A) echocardiograms of hearts; (B) HE staining; (C) Masson staining; (D) TUNEL staining. Statistical analysis of (E) ejection fraction; (F) fractional shortening; (G) collagen volume; (H) Apoptotic index; (I, J) immunoblots of Bax, Bcl2, Cleaved Caspase‐3, GAPDH and ubiquitin. (K–N) Statistical analysis of Bax/Bcl2, Cleaved Caspase‐3/GAPDH; (O) immunoblots of CaMKII, p‐CaMKII, USP10 and GAPDH; (P, Q) Statistical analysis of p‐CaMKII /CaMKII, USP10/GAPDH; The data are represented as mean ± SEM, *n* = 3–6. Statistical analysis was performed using a one‐way analysis of variance, followed by Tukey post hoc tests. (A–H, O–Q, **p < 0.05 vs. control, ^##^ *p* <  0.05 vs. DOX, ^&&^ *p* <  0.05 vs. DOX + KN‐93); (I, K, L, **p < 0.05 vs. control, ^##^ *p* <  0.05 vs. DOX); (J, M, N, **p < 0.05 vs. DOX, ^##^ *p* <  0.05 vs. DOX + KN‐93), Scale bar = 50 μm.

We initially explored the role of CaMKII and USP10 in DOX‐induced cardiotoxicity in cells. To confirm this scientific mechanism, we performed animal experiments to confirm these changes. The DOX‐induced injury model was established in C57BL/6J mice. Initially, we observed severe cardiac dysfunction and structural changes caused by DOX administration. As depicted in Figure 6, compared to the control group, the DOX group exhibited significant decreases in cardiac function parameters, namely EF and FS. Furthermore, HE and Masson's trichrome staining assays revealed increased deposition of fibrotic tissue in the DOX‐treated hearts. Meanwhile, the rate of myocardial apoptosis increased. The inhibition of CaMKII with KN‐93 mitigated cardiac dysfunction, as evidenced by increased EF, FS and improved histopathological changes. And the rate of myocardial apoptosis increased (Figure 6A–H). The alleviation of DOX‐induced cardiotoxicity through CaMKII inhibition highlights the substantial role of CaMKII in this process. After intervention with Spautin‐1, the protective effect of KN‐93 against cardiotoxicity was blocked as in the cell experiments (Figure 6A–H), These results suggest that the USP10 signaling pathway is effective in protecting the heart after CaMKII is inhibited.

### 3.7 CaMKII Inhibition With KN‐93 Alleviated DOX‐Induced Cardiac Apoptosis and Ubiquitination, and Spautin‐1 Blocked the Protective Effects

**FIGURE 7:**
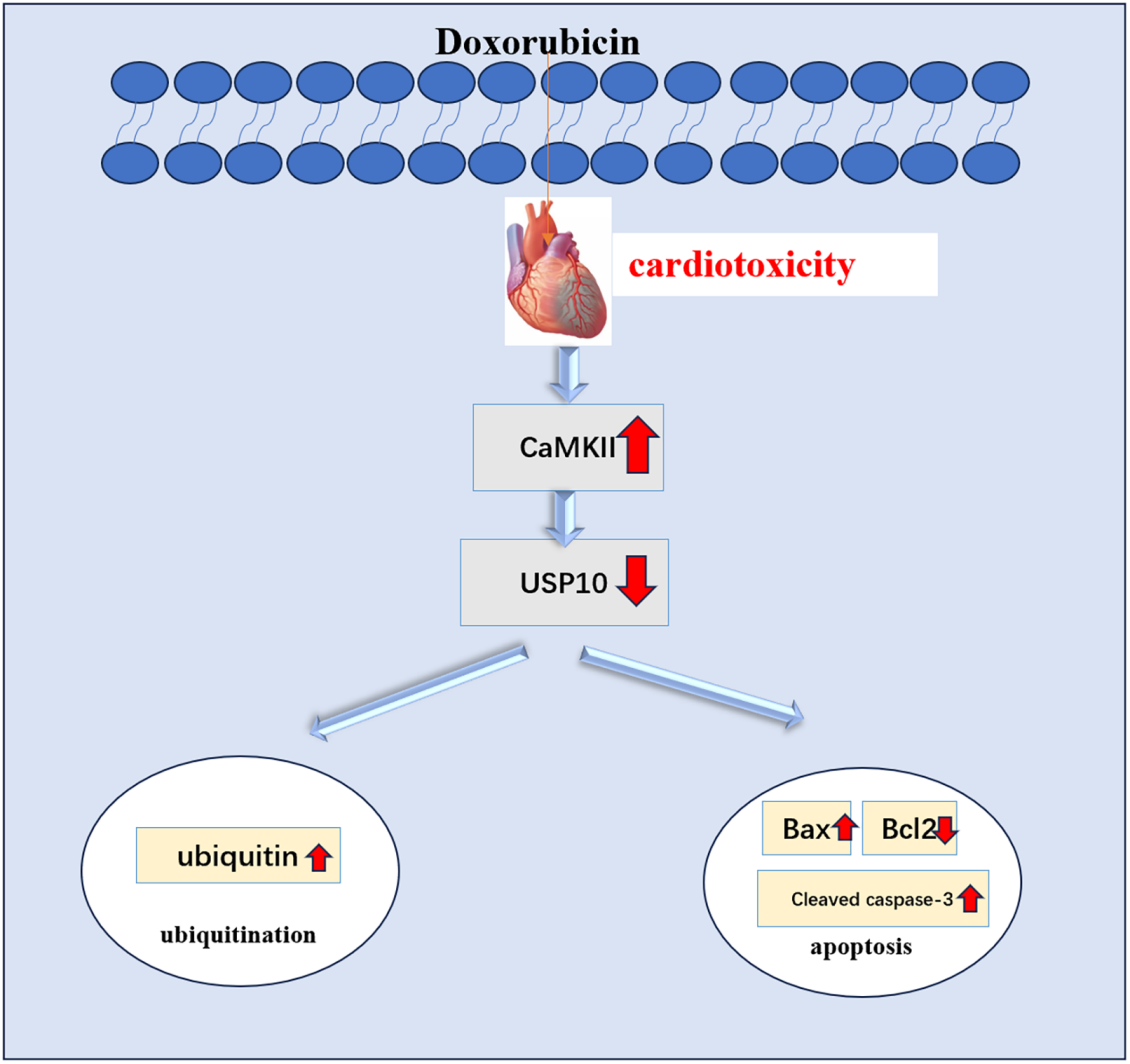
Mechanistic diagram of CaMKII exacerbates DOX‐induced cardiotoxicity by promoting ubiquitination and apoptosis through USP10 signaling inhibition.

Figure 5 depicts the substantial induction of ubiquitination and apoptosis in mice hearts following DOX administration. The expression of ubiquitin, p‐CaMKII, Bax, Cleaved Caspase‐3 and apoptotic index showed a notable increase, while those of USP10 and Bcl2 exhibited a decrease (Figure 6I–N). These findings strongly suggest the widespread presence of CaMKII in DOX‐treated hearts and establish a close relationship between ubiquitination, apoptosis, and DOX‐induced cardiotoxicity. Remarkably, the inhibition of CaMKII with KN‐93 effectively mitigated ubiquitination and apoptosis in mice hearts. Moreover, the expression levels of USP10 and Bcl2 were increased, while those of ubiquitin, p‐CaMKII, Bax, Cleaved Caspase‐3, and apoptotic index were reduced. These compelling data reinforce the pivotal role of CaMKII in DOX‐induced cardiotoxicity and underscore the potential of CaMKII inhibition as a preventive measure against DOX‐induced cardiac ubiquitination and apoptosis in mice hearts (Figure 6I–N). However, after the use of Spautin‐1 to inhibit USP10, the effect of KN‐93 on inhibiting ubiquitination and apoptosis was blocked, consistent with the findings in H9C2 cells (Figure 6O–Q). These findings confirm that the protective effect of KN‐93 on the heart was hindered after the inhibition of USP10, suggesting that the USP10 signaling pathway is an effective mechanism for protecting the heart following CaMKII inhibition (Figure 7).

## 4 Discussion

DOX, an anthracycline chemotherapy drug known for its robust and wide‐ranging anticancer properties, is essential in the management of different types of tumors [34]. Although DOX is effective in inhibiting tumor progression, its induced cardiotoxicity, as the most serious complication, worsens with increasing cumulative amounts of the administered drug, seriously jeopardizing the cardiac health of tumor patients. Therefore, researchers need to solve, at least minimize, this problem. But the etiology of DOX‐induced cardiotoxicity is intricate. DOX causes permanent damage and apoptosis in cardiomyocytes [35]. It causes myocardial fibrosis and left ventricular diastolic dysfunction [36, 37] and can progress to dilated cardiomyopathy and even end‐stage heart failure [38]. DOX‐induced cardiotoxicity is closely related to a variety of mechanisms. DOX can form a complex with tapoisomerase II and DNA in cardiomyocytes, and this ternary complex can induce DNA double‐strand formation in cardiomyocytes Disruption, leading to apoptosis is involved in DOX‐induced cardiotoxicity [39]. And studies have found that pyroptosis caused by NLRP3 inflammasomes can aggravate DOX‐induced myocardial toxicity [40], while the use of iron chelators can effectively improve DOX‐induced myocardial cell death, which confirms that ferroptosis is the main form of death in DOX‐induced myocardial injury [35]. As expected, our experiments revealed that DOX severely impaired cardiac physiological functions in mice and induced injury and apoptosis in H9C2 cardiomyocytes. These findings emphasize the substantial cardiotoxicity of DOX. The disruption of calcium homeostasis is a significant factor in the development of cardiovascular conditions. CaMKII is a crucial contributor to the progression of cardiac hypertrophy, apoptosis, and heart failure. The increased expression and phosphorylation of CaMKII can regulate intracellular Ca^2+^ balance through various pathways in the process of cardiomyocyte apoptosis. As CaMKII has been shown to be extensively involved in the pathological process of cardiovascular diseases by inducing calcium overload in cardiomyocytes leading to cellular injury, its role in cardiovascular diseases has been increasingly emphasized [41]. The exact pathways responsible for DOX‐induced cardiotoxicity remain incompletely elucidated. However, research has substantiated that the activation of CaMKII by reactive oxygen species (ROS) via oxidation plays a significant role in the development of its cardiac complications [42]. Our results showed that DOX‐induced apoptosis and increased ROS release in cardiomyocytes and mice, and CaMKII was significantly activated. In addition, we investigated the role of CaMKII in DOX‐induced cardiotoxicity by using KN‐93, which competes with calmodulin for binding to the CaMKII‐binding region and inhibits CaMKII activity. The findings from our study demonstrate that KN‐93 mitigates cardiomyocyte injury and cardiac dysfunction induced by DOX. Additionally, it suppresses apoptosis, reduces ROS levels, and exhibits a protective impact against DOX‐induced cardiotoxicity by inhibiting CaMKII to mitigate apoptosis. These results underscore the significance of CaMKII‐induced apoptosis in the development of DOX‐induced cardiotoxicity.

Ubiquitination is defined as the precise modification of a target protein by a ubiquitin molecule through a specialized series of enzymes. This process holds significant importance in various aspects of protein biology including localization, metabolism, functionality, regulation, and degradation. Furthermore, ubiquitination is intricately linked to the control of a multitude of fundamental biological processes such as cell cycle progression, proliferation, apoptosis, DNA damage repair, inflammation, immunity, and more [43]. It is worth noting that ubiquitination, like DOX, is also strongly associated with the development of tumors and cardiovascular, and is strongly associated with apoptosis [44] Through the ubiquitination of different substrates in different pathways of apoptosis, the inhibitor of apoptosis (IAP) not only inhibits the mitochondrial pathway of apoptosis, but also inhibits the exogenous pathway of apoptosis. We, therefore, speculate that ubiquitination is also closely related to DOX‐induced cardiotoxicity. Ubiquitination is a protein labeling system in cells. Different proteins are labeled with different labels, which can be recognized by other enzymatic complexes and organelles in the cell. Ubiquitination and deubiquitination is a dynamic equilibrium. The ubiquitination process usually requires the synergistic action of three ubiquitinases, of which E1 ubiquitin‐activating enzymes and E2 ubiquitin‐conjugating enzymes activate ubiquitin and link it to protein substrates, and E2 ubiquitin‐conjugating enzymes activate ubiquitin and link it to protein substrates. Under the catalysis of the three enzymes of E 1,2,3, ubiquitin is attached to the target protein or the already linked ubiquitin chain on the target protein in a specific manner. Deubiquitination is essentially a deubiquitination process of hydrolysis. Deubiquitination cleave ubiquitin molecules from the target protein substrate, thereby regulating the activity of ubiquitin ligases. The ubiquitination‐deubiquitylation pathways play a crucial role in regulating various cellular functions such as protein degradation through proteasome and lysosome pathways, apoptosis, cell survival, cell cycle progression, chromosome segregation, and gene expression. USP10, a deubiquitinase from the cysteine hydrolase family, is involved in removing ubiquitin from target proteins by cleaving the ester bond that links ubiquitin to the lysine residue of the substrate. This enzyme is essential in the ubiquitination process and significantly contributes to the regulation of cellular functions [45, 46]. Research has indicated that USP10 plays a crucial part in the advancement of tumors, encompassing tumor invasion, spread to other parts of the body, and the prediction of the disease's outcome [47, 48, 49]. In our study we found that CaMKII and apoptosis were inextricably linked in DOX‐induced cardiotoxicity, but we also found that the degree of myocardial ubiquitin expression was markedly increased, while the expression of USP10 was markedly decreased, suggesting that USP10 may play a key role in the activation of CaMKII. At the same time, we found that inhibition of CaMKII using KN‐93 resulted in a significant decrease in the degree of ubiquitin and a concomitant rise in USP10 expression. These results suggest that the regulation of ubiquitination may be an effective mechanism by which CaMKII inhibition inhibits apoptosis and alleviates DOX‐induced cardiotoxicity.

To clarify whether CaMKII exacerbates DOX‐induced cardiotoxicity by regulating USP10 to regulate ubiquitination and promote apoptosis. We used Spautin‐1, a specific inhibitor of USP10, in our experiments. As we expected, in the context of DOX‐induced H9C2 cardiomyocyte injury, the anticipated protective influence of KN‐93 on cells was negated with the introduction of Spautin‐1. This was confirmed by a notable rise in apoptosis levels, and the capacity of CaMKII inhibition to diminish ROS release was notably diminished. Most importantly, inhibition of USP10 can increase ubiquitin expression and deepen the degree of ubiquitination. The findings indicate that the suppression of USP10 intensified the level of ubiquitination, which induced apoptosis and ROS release, and activated CaMKII, resulting in the calcium overload of cardiomyocytes and aggravating the cardiotoxicity induced by DOX. To further corroborate this mechanism, we also intervened DOX mice with Spautin‐1 to observe the effects of USP10 inhibition on cardiac function in mice. KN‐93 has been shown to ameliorate cardiac impairment in DOX mice, increase EF and FS, alleviate myocardial injury and fibrosis, and inhibit apoptosis and ubiquitination in myocardium. Whereas the protective effect of KN‐93 on cardiac function in DOX mice was similarly counteracted after we used Spautin‐1 to inhibit USP10. Spautin‐1 caused a decrease in cardiac function with myocardial injury and fibrosis, while promoting apoptosis and ubiquitination in the myocardium of KN‐93‐treated mice. And as we predicted, CaMKII was also significantly activated upon inhibition of USP10. This suggests that Spautin‐1 promotes ubiquitination by inhibiting USP10, aggravates the degree of myocardial apoptosis and activates CaMKII, which aggravates cardiotoxicity in DOX mice. In addition, as Spautin‐1 also inhibits USP13 expression, we found that DOX‐induced cardiomyocyte injury also causes USP13 expression reduction, KN‐93 can increase USP13 expression, and intervention with Spautin‐1 can block the regulation of USP13 by KN‐93. Considering that USP13 and USP10 also have the role of regulating substrate deubiquitination and inhibiting the degradation of substrate ubiquitin proteasome, and are also widely involved in life processes such as cancer and cell apoptosis. In addition, USP13 can also enhance the expression of anti‐apoptotic protein Bcl‐2, thereby regulating the apoptosis signaling pathway closely related to CaMKII. These results suggest that USP13 may be a potential regulator of CaMKII, and we will explore this role in further studies.

## 5 Conclusion

Our experiments revealed that DOX‐induced myocardial apoptosis and activated CaMKII through cellular and animal levels. It also provides a new exploration of the mechanism of CaMKII in promoting apoptosis: promoting ubiquitination by inhibiting USP10. In addition, we further validated this conjecture by using the USP10‐specific inhibitor Spautin‐1, and our experiments confirmed that CaMKII regulates ubiquitination by inhibiting USP10, thereby aggravating cardiomyocyte apoptosis and further aggravating DOX‐induced cardiotoxicity. However, because the pathogenic mechanisms of CaMKII in the heart and DOX‐induced cardiac injury are complex and multifaceted, as well as Spautin‐1 has a role in inhibiting autophagy. Therefore, further studies are needed to fully unravel this complex relationship and enhance our understanding of the underlying mechanisms.

## Author Contributions

**Yitong Yang:** conceptualization (equal), data curation (equal), formal analysis (equal), investigation (equal), methodology (equal), project administration (equal), software (equal), writing – original draft (equal), writing – review and editing (equal). **Zhenyi Wang:** conceptualization (equal), data curation (equal), methodology (equal), software (equal). **Nisha Wang:** data curation (equal), formal analysis (equal), investigation (equal), methodology (equal). **Jian Yang:** conceptualization (equal). **Lifang Yang:** conceptualization (equal), data curation (equal), funding acquisition (equal), project administration (equal), resources (equal), supervision (equal), validation (equal), visualization (equal).

## Conflicts of Interest

The authors declare no conflicts of interest.

## Data Availability

The data that support the findings of this study are available on request from the corresponding.
